# Risk/benefit tradeoff of habitual physical activity and air pollution on chronic pulmonary obstructive disease: findings from a large prospective cohort study

**DOI:** 10.1186/s12916-022-02274-8

**Published:** 2022-02-28

**Authors:** Lan Chen, Miao Cai, Haitao Li, Xiaojie Wang, Fei Tian, Yinglin Wu, Zilong Zhang, Hualiang Lin

**Affiliations:** 1 Department of Epidemiology, School of Public Health, Sun Yat-sen University, Guangzhou, 510080 China; 2 Department of Social Medicine and Health Service Management, Shenzhen University General Hospital, Shenzhen, 518055 China

**Keywords:** Air pollution, Physical activity, Chronic obstructive pulmonary disease, Cohort study

## Abstract

**Background:**

The combined health impact of physical activity (PA) and air pollution on chronic obstructive pulmonary disease (COPD) remains unclear. We investigated the joint effects of habitual PA and long-term fine particulate matter (PM_2.5_) exposure on COPD incidence in a prospective population-based cohort.

**Methods:**

A prospective cohort study was conducted using data from the UK Biobank. Incidence of COPD was ascertained through linkage to the UK National Health Services register. Annual mean PM_2.5_ concentration was obtained using land use regression model. PA was measured by questionnaire and wrist-worn accelerometer. Cox proportional hazard models were applied to examine the associations between PM_2.5_, PA, and COPD. Additive and multiplicative interactions were examined.

**Results:**

A total of 266,280 participants free of COPD at baseline were included in data analysis with an average follow-up of 10.64 years, contributing to around 2.8 million person-years. Compared with participants with low level of PA, those with higher PA levels had lower risks of COPD incidence [hazard ratio (HR): 0.769, 95% CI: 0.720, 0.820 for moderate level; HR: 0.726, 95% CI: 0.679, 0.776 for high level]. By contrast, PM_2.5_ was associated with increased risk of COPD (HR per interquartile range increment: 1.065, 95% CI: 1.032, 1.099). Limited evidence of interaction between habitual PA and PM_2.5_ exposure was found. Similar results were found for accelerometer-measured PA.

**Conclusions:**

Our study suggests that habitual PA could reduce risk of COPD incidence, and such protective effects were not affected by ambient PM_2.5_ pollution exposure.

**Supplementary Information:**

The online version contains supplementary material available at 10.1186/s12916-022-02274-8.

## Background

Chronic obstructive pulmonary disease (COPD) is a leading cause of global disease burden [1]. According to the Global Burden of Diseases Study (GBD) 2017, COPD remained the most prevalent chronic respiratory disease worldwide, accounting for over 50% of chronic respiratory disease cases globally [2]. Besides, COPD has become the fifth leading cause of disability-adjusted life-years (DALYs) worldwide, responsible for approximately 81.6 million DALYs in 2017 [3].

A number of risk factors concerning the occurrence of COPD have been well documented, including genetics, smoking, occupational exposure, chronic asthma, and so on [4]. An increasing body of studies have also indicated that air pollution is an important risk factor of COPD, especially particulate matter with an aerodynamic diameter smaller than 2.5 μm (PM_2.5_) [5–7]. By contrast, physical activity (PA) has been shown as an effective measure to prevent non-communicable diseases, including COPD [8]. Exercising may increase the inhalation of air pollutants because of higher ventilation, and consequently amplify the adverse health effects of air pollutants [9]. Therefore, balancing the hazard/benefit tradeoff of air pollution and PA has become an important public issue since over 90% of the population worldwide lives in countries where air quality fails to meet the World Health Organization (WHO) guidelines [10]. Apart from inconsistency in current findings on the combined health impact of PA and air pollution [11], most previous studies solely depended on self-reported PA, which is subject to limitations of reliability and validity [12]. Recent technological advances have made wearable motion sensors, such as accelerometers, portable and convenient to use in large epidemiological studies, allowing more accurate measurement of PA with different intensities [13].

In the present study, we investigated the combined health impact of habitual PA (measured by both questionnaire and accelerometer) and chronic exposure to PM_2.5_ on COPD incidence in a prospective cohort, in an effort to bring out more solid clues for the health effects of PA on COPD with consideration of air pollution.

## Methods

### Study population

Our study applied data from the UK Biobank Cohort study [Application Number: 69550], an ongoing longitudinal cohort of over 0.5 million participants aged 40–69 years at baseline (2006–2010). The study was mainly conducted in urban areas of England, Scotland, and Wales through the UK National Health Services register [6]. All participants completed a series of baseline assessment, including socioeconomic factors, lifestyle and behavioral factors, and the history of medication and operations. Biological samples, such as blood and urine samples, were taken as well. Details of the UK Biobank protocols can be found elsewhere [14]. The UK Biobank study was approved by the North West Multi-centre Research Ethics Committee (06/MRE08/65). Informed consent was obtained from all participants [15].

**Fig. 1:**
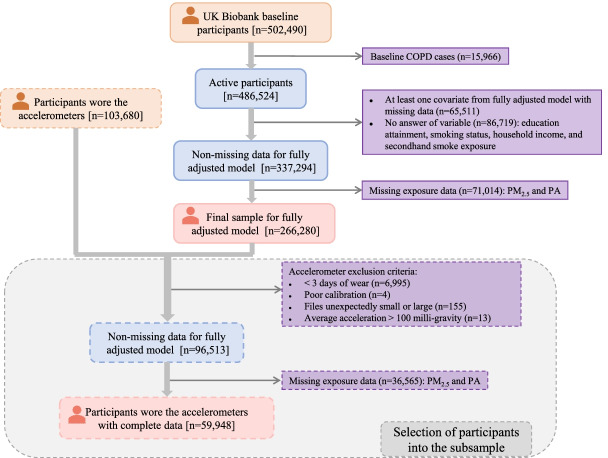
Selection of participants. Abbreviations: COPD, chronic obstructive pulmonary disease; PM_2.5_, particulate matter with an aerodynamic diameter smaller than 2.5 μm; PA, physical activity

Inclusion and exclusion procedures of the study population in the present study are outlined in Fig. 1. Of the 502,490 participants in the UK Biobank, 15,966 with prevalent COPD at baseline were excluded. We further excluded 149,230 participants due to missing data for at least one covariate in fully adjusted models, leaving 337,294 participants with complete covariate data. After further excluding 71,014 participants with missing PM_2.5_ or PA, 266,280 participants were left in our final sample. As for participants with objectively measured PA, according to data exclusion criteria of accelerometers in previous studies [16, 17] and following the same selection procedures as participants with self-reported PA, a total of 59,948 participants were left in the subsample analysis.

### Measurement of habitual physical activity (PA)

Information on habitual PA was obtained by a self-administered questionnaire and wrist-worn accelerometer. Briefly, self-reported PA was ascertained with a modified version of the International Physical Activity Questionnaire (IPAQ), which includes six questions about duration and frequency of walking, moderate-intensity and vigorous-intensity exercise undergone in the last 4 weeks [18]. Each intensity was assigned a corresponding metabolic equivalent (MET, 1 MET = 1 kilocalorie per hour per kilogram of bodyweight): 3.3 for walking, 4.0 for moderate PA, and 8.0 for vigorous PA [19]. We then quantified PA of each participant by calculating minutes of MET each week (MET-min/week) based on the reported intensity, duration, and frequency of PA in 1 week. Afterwards, participants were classified into three groups based on a standard scoring criteria of International Physical Activity Questionnaire (IPAQ) [20]: low (< 600 MET-min/week), moderate (600 to 3000 MET-min/week), and high (≥ 3000 MET-min/week). The threshold of 600 MET-min/week is equal to reaching the recommended WHO guidelines for moderate-intensity PA (150 min per week) [21].

Furthermore, we adopted a subsample of about 100,000 participants with objectively measured PA using triaxial accelerometers (Axivity AX3, Newcastle upon Tyne, UK). Participants were chosen voluntarily and accelerometers (the Axivity AX3 wrist-worn triaxial accelerometer) were sent to their personal addresses [22]. They were required to wear accelerometers on their dominant wrists for seven consecutive days. Information on the accelerometer protocol, including data extraction and processing, has been documented in detail elsewhere [23]. Individuals were grouped into three groups based on the PA tertiles [low < 24.32 milli-gravity (mg), moderate 24.32 to 30.70 mg, and high ≥ 30.70 mg] [24].

### Air pollution assessment

The annual average concentration of PM_2.5_ in 2010 was calculated using a land-use regression model (LUR) developed by the European Study of Cohorts for Air Pollution Effects (ESCAPE) project [25]. Details on the development and validation of the ESCAPE LUR models have been described elsewhere [26]. In brief, based on a range of predictive variables (such as traffic intensity, population, topography, and land use) derived from geographic information system (GIS), LUR models were used to calculate the spatial variation in air pollutant concentrations at individuals’ residential addresses provided at baseline. Leave-one-out cross-validation showed good model performance for PM_2.5_ (cross-validation *R*^2^=77%) in the southeast England area (London/Oxford) [27]. The obtained PM_2.5_ concentration in 2010 was used as a surrogate measure of long-term PM_2.5_ exposure, given the fluctuation of temporal trend of PM_2.5_ concentrations remained generally parallel during study period [28, 29].

### Outcome ascertainment

Participants were followed up from enrollment till the first occurrence of COPD, death, loss of follow-up, or 31 December 2019, whichever came first. COPD cases were identified using self-reported information, primary care data, and hospital admission data through linkage to the UK National Health Services register [18]. COPD diagnoses were coded as J40-J44 according to the International Classification of Diseases version-10 (ICD-10).

### Covariates

Potential confounders were selected based on literature review a priori [6, 11, 30], including demographic characteristics (sex, age, ethnicity, etc.), lifestyle factors (smoking status, drinking status, and fruit and vegetable intake), and so on. Details of definitions of these covariates could be seen in the Additional file 1: Supplementary methods of covariates and 4-model analytical protocol [14, 21, 31–33].

### Statistical analysis

Cox proportional hazard regression models with follow-up time as time scale were applied to examine the associations of long-term PM_2.5_ exposure and habitual PA with COPD incidence. STROBE cohort reporting guidelines were adopted as well [34]. We developed a 4-model analytical protocol by adding covariates (e.g., demographic characteristics, socioeconomic factors, lifestyle factors) incrementally. We investigated the independent associations of PA and PM_2.5_ first by building Models 1, 2, and 3. Then Model 4 was constructed for mutual adjustment. Details of the 4-model analytical protocol can be found in Additional file 1: Supplementary methods of covariates and 4-model analytical protocol.

When exposures were included as continuous variables, hazard ratio, and 95% confidence interval (HR, 95% CI) were calculated for each inter-quantile range (IQR) increment in PM_2.5_ (1.27 μg/m^3^) and per 600 MET-min/week in PA. For categorical exposures, effect estimates were calculated with reference to low level of PA or the first tertile of PM_2.5_. The proportional hazard assumption was examined by plotting Schoenfeld residuals, and no evidence of serious violation was found. Concentration-response relationship between long-term PM_2.5_ exposure and COPD incidence was examined using a spline term in Cox model, where PM_2.5_ was treated as a continuous variable with a degree of freedom (df) of 4.

Subgroup analyses stratified by PM_2.5_ tertiles (< 9.48 μg/m^3^, 9.48 to 10.27 μg/m^3^, and ≥ 10.27 μg/m^3^) or PA levels (low, moderate, and high) were also conducted using Model 3. Cochran-Armitage trend test was used to confirm the constant trend toward higher incidence of COPD with an increasing PA levels and PM_2.5_ exposure levels [35].

We further investigated the potential interactions between PA and PM_2.5_ exposure on both additive and multiplicative scales. For additive interaction, we firstly categorized study participants into three groups based on their PM_2.5_ tertiles (low < 9.48 μg/m^3^, moderate 9.48 to 10.27 μg/m^3^, and high ≥ 10.27 μg/m^3^). Combined with PA levels (low, moderate, and high), we then created a new term with nine categories representing nine combinations (3 × 3) of PM_2.5_ exposure levels and PA levels.

To test the additive interaction, we calculated the relative excess risk due to interaction (RERI) and their corresponding confidence intervals (CI) with the reference group of high level of PM_2.5_ exposure and low level of PA. RERI measures the combined excess risk in both exposed group that is due to the interaction [36]. A RERI of 0 denotes no additive interaction (i.e., the combined excess risk is the sum of their individual excess risks, and the excess risk is calculated as HR-1), a RERI of more than 0 indicates positive interaction (i.e., the combined excess risk is more than the sum of their individual excess risks), and a RERI of less than 0 implies that the combined excess risk is less than the sum of their individual excess risks [37]. For example, a negative RERI value in the present study would indicate that the benefit of PA was attenuated by residential PM_2.5_ exposure.

On multiplicative scale, we added a product term between PM_2.5_ exposure and PA levels. Likelihood tests were applied to test the significance of interaction term by comparing the model with and without the interaction term. A *p* value of the interaction term less than 0.05 indicates a multiplicative interaction [38].

For objectively measured PA data extracted from Axivity AX3, we used average acceleration to represent the overall PA intensity and categorized participants into three levels [< 24.32 milli-gravity (mg), 24.32 to 30.70 mg, and ≥ 30.70 mg]. Detailed information of accelerometer-measured PA level can be found elsewhere [24, 39]. The analyzing protocol for objectively measured PA was the same with that of the self-reported PA.

### Sensitivity analysis

We performed a number of sensitivity analyses to examine the robustness of our results. First, we additionally adjusted for baseline lung function, inverse distance to main road, and both [40, 41]. These two variables were weakly correlated with PM_2.5_ and PA (Additional file 1: Table S1). Second, instead of treating death as censored in our main analysis, we treated it as a competing risk and conducted an additional sensitivity analysis using Fine-Gray subdistribution hazards regression model [42]. Third, given the exclusion of a large number of participants with missing information, missing values of the baseline covariates were imputed with multivariate imputation via chained equation (MICE) (the number of imputations was 5) to make sure that the representativeness of the cohort was not substantially affected by exclusion of participants [18].

All analyses were conducted using R 4.0.1. A two-sided *p* value of < 0.05 was considered statistically significant.

## Results

### Baseline characteristics of participants

Table 1 presents baseline characteristics of the 266,280 participants with the mean age of 55.93 [standard deviation (SD): 8.08] years. The mean duration of follow-up was 10.64 (SD: 1.34) years. There were more females (51.15%) than males and over 95% participants were of White ethnicity. About 80% participants were classified as having moderate to vigorous PA levels. The mean annual average PM_2.5_ concentration in 2010 was higher in COPD incident cases [10.08 (SD 1.08) μg/m^3^] than in non-cases [9.93 (SD 1.04) μg/m^3^]. Detailed baseline information of the subsample with objectively measured PA was presented in Additional file 1: Table S2. The distribution of baseline characteristics was generally similar between the whole study population and the subsample.

**Table 1:** Baseline characteristics of participants in the UK Biobank Cohort

Level ^**a**^	Overall	Non-COPD case	COPD case	***P***
**Total number**	266,280	259,846	6434	–
**Follow-up duration (year)**	10.64 (1.34)	10.65 (1.33)	10.36 (1.78)	<0.001
**Age (year)**	55.93 (8.08)	55.81 (8.08)	61.16 (6.50)	<0.001
**Sex**				
Female	136,199 (51.15)	133,445 (51.36)	2754 (42.80)	<0.001
Male	130,081 (48.85)	126,401 (48.64)	3680 (57.20)	
**Ethnicity**				
Nonwhite	11,852 (4.45)	11,682 (4.50)	170 (2.64)	<0.001
White	254,428 (95.55)	248,164 (95.50)	6264 (97.36)	
**Household income (£)**				
Less than 18,000	49,790 (18.70)	47,325 (18.21)	2465 (38.31)	<0.001
18,000 to 30,999	65,324 (24.53)	63,385 (24.39)	1939 (30.14)	
31,000 to 51,999	72,779 (27.33)	71,571 (27.54)	1208 (18.78)	
52,000 to 100,000	61,171 (22.97)	60,482 (23.28)	689 (10.71)	
Greater than 100,000	17,216 (6.47)	17,083 (6.57)	133 (2.07)	
**Smoking status**				
Never	157,380 (59.10)	155,431 (59.82)	1949 (30.29)	<0.001
Previous	100,909 (37.90)	96,756 (37.24)	4153 (64.55)	
Current	7991 (3.00)	7659 (2.95)	332 (5.16)	
**BMI level**				
Normal	87,535 (32.87)	86,085 (33.13)	1450 (22.54)	<0.001
Underweight	1046 (0.39)	1015 (0.39)	31 (0.48)	
Overweight	115,818 (43.49)	113,205 (43.57)	2613 (40.61)	
Obese	61,881 (23.24)	59,541 (22.91)	2340 (36.37)	
**Intake alcohol**				
Never	17,322 (6.51)	16,753 (6.45)	569 (8.84)	<0.001
Occasional	54,536 (20.48)	53,073 (20.42)	1463 (22.74)	
Moderate	136,262 (51.17)	133,417 (51.34)	2845 (44.22)	
Heavy	58,160 (21.84)	56,603 (21.78)	1557 (24.20)	
**PA MET (minute)**	2618.74 (2640.64)	2619.42 (2635.74)	2591.50 (2831.24)	0.40
**Self-reported PA level**				
Low	49,102 (18.44)	47,607 (18.32)	1495 (23.24)	<0.001
Moderate	109,332 (41.06)	106,802 (41.10)	2530 (39.32)	
High	107,846 (40.50)	105,437 (40.58)	2409 (37.44)	
**Education attainment**				
Any school degree	103,206 (38.76)	101,080 (38.90)	2126 (33.04)	<0.001
College education	102,387 (38.45)	100,944 (38.85)	1443 (22.43)	
Vocational qualifications	16,690 (6.27)	16,082 (6.19)	608 (9.45)	
Other	43,997 (16.52)	41,740 (16.06)	2257 (35.08)	
**Fruit and vegetable intake**				
Low	71,338 (26.79)	69,503 (26.75)	1835 (28.52)	0.007
Moderate	138,311 (51.94)	135,051 (51.97)	3260 (50.67)	
High	56,631 (21.27)	55,292 (21.28)	1339 (20.81)	
**Employment status**				
Paid	167,651 (62.96)	165,175 (63.57)	2476 (38.48)	<0.001
Retired	80,759 (30.33)	77,426 (29.80)	3333 (51.80)	
Unpaid	17,870 (6.71)	17,245 (6.64)	625 (9.71)	
**Townsend deprivation index**	− 1.63 (2.86)	− 1.65 (2.85)	− 0.92 (3.19)	<0.001
**PM**_**2.5**_ **(μg/m**^**3**^**)**	9.94 (1.04)	9.93 (1.04)	10.08 (1.08)	<0.001

*Abbreviations*: *COPD* chronic obstructive pulmonary diseases, *BMI* body mass index, *PA* physical activity, *PM*_*2.5*_ particulate matter with an aerodynamic diameter < 2.5 μm, *MET* metabolic equivalents

^a^The statistics are shown as mean [standard deviation (SD)] for continuous variables and number (%) for categorical variables

### Association between self-reported PA and COPD incidence

Table 2 shows the associations between self-reported PA and incident COPD. Higher PA level was associated with a lower incidence of COPD. The associations were robust to adjustment for various covariates, including PM_2.5_. In Model 4, compared with low PA level, the HR (95% CI) of developing COPD was 0.769 (95% CI: 0.720, 0.820) and 0.726 (95% CI: 0.679, 0.776) for moderate and high level of PA, respectively. In subgroup analysis, the negative association of self-reported PA and COPD incidence remained stable in participants exposed to different PM_2.5_ levels (Table 3).

**Table 2:** Associations of habitual physical activity (PA) and long-term PM_2.5_ exposure and COPD incidence (*n* = 6381) in UK Biobank Cohort

Levels ^**a**^		Model l ^**b**^	Model 2 ^**b**^	Model 3 ^**b**^	Model 4 ^**b**^
		HR (95% CI)	HR (95% CI)	HR (95% CI)	HR (95% CI)
**Self-reported PA**	**Case**				
Low	1495	1.000	1.000	1.000	1.000
Moderate	2530	**0.758 (0.711, 0.809)**	**0.761 (0.713, 0.812)**	**0.769 (0.721, 0.820)**	**0.769 (0.720, 0.820)**
High	2409	**0.752 (0.705, 0.803)**	**0.713 (0.668, 0.761)**	**0.725 (0.678, 0.775)**	**0.726 (0.679, 0.776)**
*P* for trend	–	**<0.0001**	**<0.0001**	**<0.0001**	**<0.0001**
Per 600 MET-min/week increment	6434	**0.990 (0.985, 0.996)**	**0.986 (0.980, 0.991)**	**0.987 (0.981, 0.993)**	**0.987 (0.982, 0.993)**
**Objectively measured PA**					
Low	253	1.000	1.000	1.000	1.000
Moderate	434	**0.676 (0.589, 0.777)**	**0.754 (0.655, 0.867)**	**0.757 (0.657, 0.871)**	**0.757 (0.658, 0.872)**
High	403	**0.542 (0.462, 0.636)**	**0.637 (0.540, 0.752)**	**0.635 (0.538, 0.750)**	**0.635 (0.538, 0.750)**
*P* for trend	–	**<0.0001**	**<0.0001**	**<0.0001**	**<0.0001**
Per IQR increment	1090	**0.658 (0.602, 0.72)**	**0.741 (0.677, 0.812)**	**0.743 (0.678, 0.814)**	**0.743 (0.679, 0.814)**
**PM**_**2.5**_ **levels**					
Low	1959	1.000	1.000	1.000	1.000
Moderate	2164	**1.215 (1.143, 1.292)**	1.059 (0.995, 1.127)	1.052 (0.989, 1.119)	1.050 (0.987, 1.117)
High	2311	**1.448 (1.363, 1.538)**	**1.094 (1.025, 1.167)**	**1.072 (1.005, 1.144)**	**1.068 (1.001, 1.140)**
*P* for trend	–	**<0.0001**	**0.007**	**0.036**	**0.046**
Per IQR increment (1.27 μg/m^3^)	6434	**1.243 (1.209, 1.278)**	**1.080 (1.046, 1.115)**	**1.067 (1.034, 1.101)**	**1.065 (1.032, 1.099)**

The bold type represents the statistically significant differences (*p* < 0.05)

*Abbreviations*: *PM*_*2.5*_ particulate matter with aerodynamic diameter < 2.5 μm, *HR* hazard ratio, *CI* confidence interval, *PA* physical activity, *COPD* chronic obstructive pulmonary disease

^a^: PM_2.5_ exposure levels (low, moderate, and high) were defined by PM_2.5_ tertiles (< 9.48 μg/m^3^, 9.48 to 10.27 μg/m^3^, and ≥ 10.27 μg/m^3^), self-reported PA levels were defined according to a standard scoring criteria of International Physical Activity Questionnaire (IPAQ): low (< 600 MET-min/week), moderate (600 to 3000 MET-min/week), and high (≥ 3000 MET-min/week), and objectively measured PA levels were defined by objectively measured PA tertiles [< 24.32 milli-gravity (mg), 24.32 to 30.70 mg, and ≥ 30.70 mg]

^b^ Model 1 was adjusted for age at enrolment, sex, and ethnicity. Model 2 was further adjusted for household income, employment status, education, and Townsend deprivation index. Model 3 was further adjusted for lifestyle factors (smoking status, alcohol intake frequency, fruit and vegetable intake), body mass index (BMI), and secondhand smoke exposure. And Model 4 was further mutually adjusted by PA (effect estimation of PM_2.5_) or PM_2.5_ (effect estimation of PA)

**Table 3:** Associations between habitual PA levels and COPD incidence when exposed to different PM_2.5_ levels

PA levels ^**a**^		Low-level PM_**2.5**_		Moderate-level PM_**2.5**_		High-level PM_**2.5**_	
	Case	HR (95% CI) ^**b**^	***P***	HR (95% CI) ^**b**^	***P***	HR (95% CI) ^**b**^	***P***
**Self-reported PA**							
Low	1495	1.000	–	1.000	–	1.000	–
Moderate	2530	**0.786 (0.698, 0.884)**	**<0.0001**	**0.727 (0.65, 0.812)**	**<0.0001**	**0.792 (0.711, 0.882)**	**<0.0001**
High	2409	**0.714 (0.633, 0.806)**	**<0.0001**	**0.707 (0.631, 0.792)**	**<0.0001**	**0.751 (0.672, 0.84)**	**<0.0001**
Trend test	–		**<0.0001**		**<0.0001**		**<0.0001**
**Objectively measured PA**							
Low	253	1.000	–	1.000	–	1.000	–
Moderate	434	0.835 (0.651, 1.072)	0.157	**0.696 (0.549, 0.883)**	**0.003**	**0.752 (0.587, 0.962)**	**0.024**
High	403	**0.656 (0.489, 0.881)**	**0.005**	**0.571 (0.429, 0.76)**	**<0.0001**	**0.696 (0.523, 0.927)**	**0.013**
Trend test	–		**0.005**		**0.007**		**0.001**

The bold type represents the statistically significant differences (*p* < 0.05)

*Abbreviations*: *PM*_*2.5*_ particulate matter with aerodynamic diameter < 2.5 μm, *HR* hazard ratio, *CI* confidence interval, *PA* physical activity, *COPD* chronic obstructive pulmonary diseases

^a^ Self-reported PA levels were defined according to a standard scoring criteria of International Physical Activity Questionnaire (IPAQ): low (< 600 MET-min/week), moderate (600 to 3000 MET-min/week), and high (≥ 3000 MET-min/week), and objectively measured PA levels were defined by objectively measured PA tertiles [< 24.32 milli-gravity (mg), 24.32 to 30.70 mg, and ≥ 30.70 mg]

^b^All results were calculated fully adjusted by covariates in Model 3: age at enrolment, sex, ethnicity, household income, employment status, education, Townsend deprivation index, smoking status, alcohol intake frequency, fruit and vegetable intake, body mass index (BMI) and secondhand smoke exposure

### Association between chronic PM_2.5_ exposure and COPD incidence

In contrast to PA, positive associations of PM_2.5_ with COPD incidence were observed, and the associations remained unchanged after adjustment for PA and other covariates (Table 2). An IQR increment in PM_2.5_ was associated with a 6.5% (95% CI: 3.2%, 9.9%) increase in COPD risk. In tertile-based analysis, compared with participants exposed to low level of PM_2.5_, the HR of having COPD was 1.050 (95% CI: 0.987, 1.117) and 1.068 (95% CI: 1.032, 1.140) for those exposed to moderate and high levels of PM_2.5_, respectively. The concentration–response curve for the association between PM_2.5_ and COPD is presented in Additional file 1: Figure S1. The positive associations remained in subgroups with different PA levels, though some were statistically non-significant (Table 4).

**Table 4:** Associations between PM_2.5_ levels and COPD incidence when exposed to different self-reported physical activity (PA) levels

PM_**2.5**_ levels ^**a**^		Low-level PA		Moderate-level PA		High-level PA	
	Case	HR (95% CI) ^**b**^	***P***	HR (95% CI) ^**b**^	***P***	HR (95% CI) ^**b**^	***P***
Low	1959	1.000	–	1.000	–	1.000	–
Moderate	2164	1.071 (0.942, 1.218)	0.296	1.004 (0.909, 1.109)	0.940	1.084 (0.980, 1.199)	0.117
High	2311	1.053 (0.920, 1.206)	0.450	1.048 (0.945, 1.163)	0.370	1.099 (0.989, 1.222)	0.081
Trend test	–		0.464		0.364		0.081

*Abbreviations*: *HR* hazard ratio, *CI* confidence interval, *PA* physical activity, *COPD* chronic obstructive pulmonary disease, *PM*_*2.5*_ particulate matter with aerodynamic diameter < 2.5 μm

^a^ PM_2.5_ exposure levels (low, moderate, and high) were defined by PM_2.5_ tertiles (< 9.48 μg/m^3^, 9.48 to 10.27 μg/m^3^, and ≥ 10.27 μg/m^3^), and self-reported PA levels were defined according to a standard scoring criteria of International Physical Activity Questionnaire (IPAQ): low (< 600 MET-min/week), moderate (600 to 3000 MET-min/week), and high (≥ 3000 MET-min/week)

^**b**^ All results were calculated fully adjusted by covariates in Model 3: age at enrolment, sex, ethnicity, household income, employment status, education, Townsend deprivation index, smoking status, alcohol intake frequency, fruit and vegetable intake, body mass index (BMI), and secondhand smoke exposure

### Potential interaction between self-reported PA and PM_2.5_ on the COPD incidence

Table 5 shows the combined health impact of self-reported PA level and chronic exposure to PM_2.5_ on COPD incidence. Participants with higher PA or lower PM_2.5_ exposure generally had lower risk of COPD. Using the participants with low level of PA and high PM_2.5_ exposure as the reference, those with high PA level and low PM_2.5_ exposure had the lowest risk of developing COPD (HR: 0.681, 95% CI: 0.607, 0.764).

**Table 5:** Combined effects of habitual physical activity, long-term PM_2.5_ exposure, and COPD incidence in the UK Biobank

PA levels ^**a**^	PM_**2.5**_ levels (HR, 95% CI) ^a, ^^**b**^			RERI ^**c**^		***P*** for interaction ^**d**^
	High	Moderate	Low	Moderate PM_2.5_ level	Low PM_2.5_ level	
**Self-reported PA**						0.805
Low	1.000	1.026 (0.909, 1.159)	0.955 (0.840, 1.085)			
Moderate	**0.792 (0.711, 0.881)**	**0.753 (0.674, 0.840)**	**0.747 (0.667, 0.836)**	− 0.01 (− 0.18, 0.17)	0.02 (− 0.14, 0.19)	
High	**0.745 (0.668, 0.831)**	**0.736 (0.658, 0.822)**	**0.681 (0.607, 0.764)**	0.01 (− 0.16, 0.18)	0.02 (− 0.15, 0.19)	
**Objectively measured PA**						0.998
Low	1.000	1.088 (0.885, 1.337)	0.923 (0.740, 1.152)			
Moderate	**0.755 (0.592, 0.963)**	0.790 (0.620, 1.006)	0.735 (0.573, 0.943)	− 0.05 (− 0.35, 0.25)	0.06 (− 0.23, 0.34)	
High	**0.693 (0.527, 0.911)**	**0.653 (0.492, 0.866)**	**0.574 (0.432, 0.764)**	− 0.13 (− 0.44, 0.18)	0.02 (− 0.14, 0.19)	

*Abbreviations*: *HR* hazard ratio, *CI* confidence interval, *RERI* relative excess risk due to interaction, *PA* physical activity, *PM*_*2.5*_ particulate matter with aerodynamic diameter < 2.5 μm, *COPD* chronic obstructive pulmonary disease

^a^: PM_2.5_ exposure levels (low, moderate, and high) were defined by PM_2.5_ tertiles (< 9.48 μg/m^3^, 9.48 to 10.27 μg/m^3^, and ≥10.27 μg/m^3^), self-reported PA levels were defined according to a standard scoring criteria of International Physical Activity Questionnaire (IPAQ): low (< 600 MET-min/week), moderate (600 to 3000 MET-min/week), and high (≥3000 MET-min/week), and objectively measured PA levels were defined by objectively measured PA tertiles (< 24.32 milli-gravity (mg), 24.32 to 30.70 mg, and ≥ 30.70 mg)

^b^All results were calculated based on covariates in Model 3: age at enrolment, sex, ethnicity, BMI, education, household income, employment status, smoking status, alcohol drinking, fruit and vegetable intake, secondhand smoke exposure, and Townsend deprivation index

^c^ The estimates of RERI were calculated based on the reference group with high level of PM_2.5_ exposure and low level of PA

^d^ Likelihood tests were applied to test the significance of interaction term by comparing the model with and without the interaction term

Results of interaction between self-reported PA and PM_2.5_ on the incidence of COPD are presented in Additional file 1: Table S3, Table S4. On additive scale, little evidence of interaction was found (Additional file 1: Table S3). Similar results were also observed on multiplicative scale, with all *p* values of the interaction term > 0.05 (Additional file 1: Table S4).

### Results of objectively measured PA and COPD incidence

Overall, similar results were found in analyses among the subsample with objectively measured PA. Higher level of objectively measured PA was associated with lower risk of COPD incidence (Table 2), and the negative association remained across different PM_2.5_ levels (Table 3). Participants with high PA level and low PM_2.5_ exposure had the lowest risk of developing COPD (HR: 0.574, 95% CI: 0.432, 0.764) with reference to those with low level of PA and high level of PM_2.5_ exposure (Table 5). No interaction between objectively measured PA and PM_2.5_ were found, either on additive or multiplicative scale (Additional file 1: Table S3, Table S4).

### Sensitivity analyses

The results of sensitivity analyses are presented in Additional file 1: Table S3-S9. Additional adjustment for baseline lung function or inverse distance to main road did not alter the results materially, especially for PA (Additional file 1: Table S3-S6). The associations between PA, long-term PM_2.5_ exposure, and incident COPD also remained robust when treating all-cause death as a competing risk (Additional file 1: Table S7). Furthermore, after the imputation of baseline missing data, the effect estimates of the associations between PA and long-term PM_2.5_ exposure and COPD incidence were similar as well (Additional file 1: Table S8, Table S9).

## Discussion

To the best of our knowledge, this is the largest prospective cohort study so far to investigate the independent and combined health effects of habitual PA (measured by both questionnaire and accelerometer) and long-term PM_2.5_ exposure on the COPD incidence in adults. Higher level of habitual PA was significantly associated with lower risk of COPD incidence, regardless of the level of PM_2.5_ concentration. By contrast, positive associations between long-term PM_2.5_ exposure and COPD were observed. Little evidence of interaction between habitual PA and PM_2.5_ exposure on COPD development was observed, either on additive or multiplicative scale.

PA is a well-recognized protective factor against COPD [43, 44]. Consistent with previous findings [31, 45], we observed that higher levels of PA, both self-reported and objectively measured, were associated with a lower risk of COPD incidence. Another analysis using the UK Biobank data also reported a negative association between self-reported PA and COPD hospital admission (HR: 0.70, 95% CI: 0.66, 0.75) [6]. According to previous studies, regular PA could reduce the risk of many adverse health outcomes partly because of its anti-inflammatory effect on the inflammatory process [46, 47] .

The adverse health effects of exposure to PM_2.5_ on the development of COPD have been well documented [48–50]. Similarly, we also observed significant associations between exposure to PM_2.5_ and increased risk of COPD. Consistent with our findings, Doiron et al. reported that higher concentrations of PM_2.5_ was associated with increased COPD prevalence (OR: 1.52, 95% CI: 1.42, 1.62) based on per 5 μg/m^3^ increment [6]. However, inconsistent results still remain. For example, in a large multi-country study, the ESCAPE (European Studies on Chronic Air Pollution Effects) study, no significant associations of PM_2.5_ with longitudinal change in lung function was observed [51]. Difference in health indicator may explain the heterogeneity to some extent: we focused on the incidence of COPD, while Adam et al. concentrated on the change in lung function metrics. Apart from the heterogeneity in study period, population, and location, the reasons behind the inconsistent findings require further investigations.

In analysis of combined effects of habitual PA and PM_2.5_ on COPD incidence, the negative associations between PA and COPD remained stable regardless of PM_2.5_ levels, which was in line with Kubesch et al.’s findings. Kubesch et al. [52] reported that, even in an environment with higher air pollution, intermittent moderate PA had beneficial effects on pulmonary function in a healthy population. Consistently, two studies based on the Danish Diet, Cancer, and Health Cohort yielded similar findings [53, 54], as both of them reported that the benefits of PA against asthma/COPD hospitalization were not attenuated by air pollution. For other health outcomes, two recent studies [11, 55] also reported independent associations of PA and air pollution with mortality or life expectancy. However, some other studies reported significant, but controversial results of the interactions between PA and air pollution on respiratory diseases. For example, in a cohort study in Taiwan [10], negative interactions between habitual PA and long-term exposure to PM_2.5_ were observed, as the beneficial health effects of PA on lung function were reportedly decreased by ambient PM_2.5_ exposure. By contrast, Matt et al. [56] reported that a one unit [1% heart rate max (HRmax)] increase of PA was observed to reduce the immediate negative effects of particulate matter (PM_2.5_) upon peak expiratory flow (0.02 L/min). Similarly, Toledo et al. [57] suggested that regular moderate-intensity aerobic physical training attenuated the development of pulmonary diseases induced by cigarette smoke exposure. It is difficult to directly compare our results with the previous studies since the study period, targeted population, health indicators, and PA measurement varied. Further studies are warranted to better characterize the potential interactions between PA and air pollution.

The mechanisms underlying the potential interaction between PA and PM_2.5_ are currently unclear. Previous studies suggested that exercise of moderate intensity may improve immune responses to lower chronic low-grade inflammation and improve a variety of immune markers in several disease conditions [58, 59]. There was also evidence suggesting that exposure to higher levels of air pollution could lead to declines in immunity stability through oxidative stress and chronic inflammation response [60]. The benefits of habitual PA may not be counteracted by the short-term elevated PM_2.5_ exposure during exercise, especially in areas where PM_2.5_ level are low to moderate, such as the UK [61]. Furthermore, the additional inhaled air pollutants due to PA only constitute a small fraction of the total inhaled air pollutants [62], which may not induce serious impairment on the respiratory system.

### Strengths and limitations of the study

Our study has several important strengths. First, we used a large cohort of 266,280 participants with extensive information on a wide range of potential confounders, which enabled us to investigate the associations more reliably. More importantly, in addition to self-reported PA, we did an additional analysis in a subsample of nearly 100,000 participants who wore Axivity AX3 triaxial accelerometers to measure their daily PA duration and volume [24], and similar results were found. Such objective method provided more accurate measurements, which is a main challenge in studies using self-reported PA [13].

Our study also has some limitations. First, we only used the annual average PM_2.5_ concentration in 2010 as a proxy for the long-term PM_2.5_ exposure, which may have led to exposure misclassification. However, many previous studies suggested that the spatial distribution of PM_2.5_ generally remains stable in the same region over a period as long as > 10 years [27, 29, 63]. In the UK, the annual average concentration of PM_2.5_ between 2010 and 2019 were relatively stable according to the Department for Environment Food & Rural Affairs of the UK (https://www.gov.uk/government/statistics/emissions-of-air-pollutants/emissions-of-air-pollutants-in-the-uk-summary). Therefore, the annual concentration of PM_2.5_ in 2010 could serve as a surrogate measure of long-term exposure over the study period. Despite the issue mentioned above, another potential limitation of the study is the exclusion of a relatively large number of participants due to missing data for covariates, which generated a relatively healthier cohort [participants in the analytical cohort were more likely to be younger and physically active (Additional file 1: Table S10)]. However, the difference was not substantial, especially the difference in PM_2.5_ (as small as 0.13 μg/m^3^), and it should not seriously bias our results. Furthermore, as an alternative approach, we additionally conducted multiple imputations for those missing covariates and the associations of PA and long-term PM_2.5_ exposure with COPD incidence remained stable (Additional file 1: Table S8 and Table S9). In addition, compared with the general population, the COPD prevalence was lower in the UK Biobank cohort [the UK Biobank: 0.4% for men, and 0.4% for women; the UK general population: 3% for men, and 2% for women among participants between 55 and 64 years], which implies the evidence of a healthy volunteer bias [64]. However, it has been suggested that this may not influence the valid estimates of associations [6] since sufficiently large numbers of individuals with different levels of exposures were investigated with high internal validity [64], but may only affect the extrapolation and underestimate the associations of PM_2.5_ and COPD incidence in a general population [6]. Last, our study is based on a European cohort with a lower PM_2.5_ concentration [65], findings of our study may therefore not be generalizable to populations in areas with relatively high PM_2.5_ concentrations.

## Conclusions

In conclusion, based on a large cohort study, we found that long-term exposure to PM_2.5_ was associated with higher risk of COPD incidence. By contrast, both self-reported and objectively measured PA were associated with lower risk of COPD regardless of the levels of PM_2.5_, indicating independent effects of PA and PM_2.5_ on COPD incidence.

## Supplementary Information

**Additional file 1: **Supplementary methods of covariates and 4-model analysis protocol. **Table S1.** Correlation between exposure variables and covariates (Spearman correlation coefficients). **Table S2.** Baseline information of the subsample with objectively measured PA. **Table S3.** Relative excess risk due to interaction (RERI, 95% CI) of long-term PM_2.5_ exposure and habitual PA levels. **Table S4.** Combined effects of long-term PM_2.5_ exposure levels and physical activity (PA) levels on COPD incidence on multiplicative scales. **Table S5.** Associations of PM_2.5_ exposure and COPD incidence adjusted by baseline lung function, inverse distance to major road, and both. **Table S6.** Combined effects of long-term PM_2.5_ exposure and self-reported physical activity (PA) on COPD incidence. **Table S7.** Associations between self-reported PA, long-term PM_2.5_ exposure and COPD incidence by treating all-cause death as a competing risk. **Table S8.** Associations between self-reported PA, long-term PM_2.5_ exposure and COPD incidence after conducting multiple imputations for missing covariates. **Table S9.** Interaction between PA and long-term PM_2.5_ exposure on both additive and multiplicative scales after conducting multiple imputations for missing covariates (*N*=357,603). **Table S10.** Baseline characteristics of the excluded and included participants. **Figure S1.** Concentration-response relationship between long-term PM_2.5_ exposure and COPD incidence.

## Data Availability

The datasets generated and analyzed during the current study are available upon reasonable request to the Access Management System (AMS) through the UK Biobank website (https://www.ukbiobank.ac.uk/enable-your-research/apply-for-access).
