# Supplementary material for: Risk/benefit tradeoff of habitual physical activity and air pollution on chronic pulmonary obstructive disease: findings from a large prospective cohort study
Source: BMC Med. 2022 Feb 28;20:70. doi: 10.1186/s12916-022-02274-8 (PMC8883705; doi:10.1186/s12916-022-02274-8)
Supplement: Supplementary file 1 — Additional file 1: Supplementary methods of covariates and 4-model analysis protocol. Table S1. Correlation between exposure variables and covariates (Spearman correlation coefficients). Table S2. Baseline information of the subsample with objectively measured PA. Table S3. Relative excess risk due to interaction (RERI, 95% CI) of long-term PM2.5 exposure and habitual PA levels. Table S4. Combined effects of long-term PM2.5 exposure levels and physical activity (PA) levels on COPD incidence on multiplicative scales. Table S5. Associations of PM2.5 exposure and COPD incidence adjusted by baseline lung function, inverse distance to major road, and both. Table S6. Combined effects of long-term PM2.5 exposure and self-reported physical activity (PA) on COPD incidence. Table S7. Associations between self-reported PA, long-term PM2.5 exposure and COPD incidence by treating all-cause death as a competing risk. Table S8. Associations between self-reported PA, long-term PM2.5 exposure and COPD incidence after conducting multiple imputations for missing covariates. Table S9. Interaction between PA and long-term PM2.5 exposure on both additive and multiplicative scales after conducting multiple imputations for missing covariates (N=357,603). Table S10. Baseline characteristics of the excluded and included participants. Figure S1. Concentration-response relationship between long-term PM2.5 exposure and COPD incidence. [file 12916_2022_2274_MOESM1_ESM.docx]

Additional File 1

Supplementary methods of covariates and 4-model analytical protocol

Covariates adjusted in analysis models:

Potential confounders were selected mainly based on literature review, including age at recruitment (strata variable, 5-year interval), sex (strata variable, male and female), ethnicity (strata variable, White and non-White), body mass index (BMI) [underweight (< 18.5 kg/m^2^), normal (18.5 to 25 kg/m^2^), overweight (25 to 30 kg/m^2^), and obese (≥ 30 kg/m^2^)], smoking status (never, previous, and current), drinking status (never, occasional, moderate, and heavy), fruit and vegetable intake (low, moderate, and high), education (college education, any school degree, vocational degree, and other), and secondhand smoke exposure (smoke at home and outside home). In addition, annual household income (< 18,000￡, 18,000 to 30,999￡, 31,000 to 51,999￡, 52,000 to 100,000￡, > 100,000￡) and Townsend deprivation index (calculated according to area-level unemployment, non-car and non-home ownership, and household overcrowding) were included as individual and neighborhood-level economic level, respectively. Details of these measurements can be found elsewhere.

4-model analytical protocol:

We developed a 4-model analysis protocol by adding covariates (e.g., demographic characteristics, socioeconomic factors, lifestyle factors, etc.) incrementally. We investigated the independent associations of PA and PM_2.5_ first by building Model 1, 2 and 3. Then Model 4 was constructed for mutual adjustment. Model 1 was adjusted for age recruited in cohort, sex, and ethnicity. Model 2 was further adjusted for household income, employment status, education, and Townsend deprivation index. Model 3 was further adjusted for lifestyle factors (smoking status, alcohol intake frequency, fruit and vegetable intake), body mass index (BMI), and secondhand smoke exposure. And Model 4 was further mutually adjusted by PA (effect estimation of PM_2.5_) or PM_2.5_ (effect estimation of PA).

Table S1. Correlation between exposure variables and covariates (Spearman correlation coefficients)

|  | **PM_2.5_** | **PA** | **FEV_1_** |
| --- | --- | --- | --- |
| **PA** | -0.010 |  |  |
| **FEV_1_** | -0.012 | 0.032 |  |
| **Inverse distance to main road** | 0.436 | -0.006 | -0.003 |

Abbreviations: FEV_1_, forced expiratory volume in 1 second; PA, physical activity; PM_2.5_, particulate matter with aerodynamic diameter < 2.5 µm.

Table S2. Baseline information of the subsample with objectively-measured PA

| **Variable *** | **Overall** | **Non-COPD case** | **COPD case** | ***P*** |
| --- | --- | --- | --- | --- |
| **Total number** | 59,948 | 58,858 | 1,090 |  |
| **Follow-up duration (year)** | 10.74 (0.89) | 10.74 (0.88) | 10.55 (1.08) | <0.0001 |
| **Age (year)** | 55.77 (7.83) | 55.68 (7.82) | 60.91 (6.24) | <0.0001 |
| **Sex** |  |  |  |  |
| Female | 32,002 (53.38) | 31,515 (53.54) | 487 (44.68) | <0.0001 |
| Male | 27,946 (46.62) | 27,343 (46.46) | 603 (55.32) |  |
| **Ethnicity** |  |  |  |  |
| Nonwhite | 1,747 (2.91) | 1,725 (2.93) | 22 (2.02) | 0.092 |
| White | 58,201 (97.09) | 57,133 (97.07) | 1,068 (97.98) |  |
| **Household income (￡)** |  |  |  |  |
| Less than 18,000 | 7,751 (12.93) | 7,462 (12.68) | 289 (26.51) | <0.0001 |
| 18,000 to 30,999 | 13,880 (23.15) | 13,555 (23.03) | 325 (29.82) |  |
| 31,000 to 51,999 | 17,393 (29.01) | 17,113 (29.08) | 280 (25.69) |  |
| 52,000 to 100,000 | 16,061 (26.79) | 15,900 (27.01) | 161 (14.77) |  |
| Greater than 100,000 | 4,863 (8.11) | 4,828 (8.20) | 35 (3.21) |  |
| **Smoking status** |  |  |  |  |
| Never | 35,670 (59.50) | 35,328 (60.02) | 342 (31.38) | <0.0001 |
| Previous | 22,815 (38.06) | 22,109 (37.56) | 706 (64.77) |  |
| Current | 1,463 (2.44) | 1,421 (2.41) | 42 (3.85) |  |
| **BMI level** |  |  |  |  |
| Normal | 23,254 (38.79) | 22,956 (39.00) | 298 (27.34) | <0.0001 |
| Underweight | 282 (0.47) | 278 (0.47) | 4 (0.37) |  |
| Overweight | 25,079 (41.83) | 24,627 (41.84) | 452 (41.47) |  |
| Obese | 11,333 (18.90) | 10,997 (18.68) | 336 (30.83) |  |
| **Intake alcohol** |  |  |  |  |
| Never | 3,057 (5.10) | 2,969 (5.04) | 88 (8.07) | <0.0001 |
| Occasional | 11,462 (19.12) | 11,257 (19.13) | 205 (18.81) |  |
| Moderate | 31,176 (52.01) | 30,680 (52.13) | 496 (45.50) |  |
| Heavy | 14,253 (23.78) | 13,952 (23.70) | 301 (27.61) |  |
| **Average PA volume (mg)** | 28.34 (8.23) | 28.41 (8.22) | 24.83 (7.70) | <0.0001 |
| **PA level** |  |  |  |  |
| Low | 19,009 (31.71) | 18,466 (31.37) | 543 (49.82) | <0.0001 |
| Moderate | 20,155 (33.62) | 19,829 (33.69) | 326 (29.91) |  |
| High | 20,784 (34.67) | 20,563 (34.94) | 221 (20.28) |  |
| **Education attainment** |  |  |  |  |
| Any school degree | 22,153 (36.95) | 21,754 (36.96) | 399 (36.61) | <0.0001 |
| College education | 28,289 (47.19) | 27,930 (47.45) | 359 (32.94) |  |
| Vocational qualifications | 3,045 (5.08) | 2,950 (5.01) | 95 (8.72) |  |
| Other | 6,461 (10.78) | 6,224 (10.57) | 237 (21.74) |  |
| **Fruit & vegetable intake** |  |  |  |  |
| Low | 14,817 (24.72) | 14,535 (24.70) | 282 (25.87) | 0.489 |
| Moderate | 32,351 (53.97) | 31,782 (54.00) | 569 (52.20) |  |
| High | 12,780 (21.32) | 12,541 (21.31) | 239 (21.93) |  |
| **Employment status** |  |  |  |  |
| Paid | 38,814 (64.75) | 38,338 (65.14) | 476 (43.67) | <0.0001 |
| Retired | 17,614 (29.38) | 17,085 (29.03) | 529 (48.53) |  |
| Unpaid | 3,520 (5.87) | 3,435 (5.84) | 85 (7.80) |  |
| **Townsend deprivation index** | -1.82 (2.74) | -1.83 (2.74) | -1.24 (2.99) | <0.0001 |
| **PM_2.5_ (ug/m^3^)** | 9.88 (1.03) | 9.88 (1.03) | 9.98 (0.99) | 0.002 |

*****: The statistics are shown as mean [standard deviation (SD)] for continuous variables and number (%) for categorical variables.

Abbreviations: COPD, chronic obstructive pulmonary diseases; BMI, body mass index; PA, physical activity; PM_2.5_, particulate matter with an aerodynamic diameter < 2.5 μm; MET, metabolic equivalents; mg, milli-gravity.

Table S3. Relative excess risk due to interaction (RERI, 95% CI) of long-term PM_2.5_ exposure and habitual PA levels

|  | **Mod 11** * | **Mod 12** * | **Mod 21** * | **Mod 22** * |
| --- | --- | --- | --- | --- |
| **Self-reported PA †** | -0.01 (-0.18, 0.17) | 0.01 (-0.16, 0.18) | 0.02 (-0.14, 0.19) | 0.02 (-0.15, 0.19) |
| **Adjusted by both † ‡** | -0.01 (-0.18, 0.17) | 0.01 (-0.16, 0.18) | 0.02 (-0.14, 0.19) | 0.02 (-0.15, 0.19) |
| **Objectively-measure PA †** | -0.05 (-0.35, 0.25) | -0.13 (-0.44, 0.18) | 0.06 (-0.23, 0.34) | -0.04 (-0.33, 0.25) |

**†**: Cox proportional hazard model adjusted by age at enrolment, sex, ethnicity, household income, employment status, education, Townsend deprivation index, smoking status, alcohol intake frequency, fruit and vegetable intake, body mass index (BMI) and secondhand smoke exposure.

**‡**: Cox proportional hazard model was further adjusted by baseline lung function and inverse distance to main road.

*: The estimates were calculated based on the reference group with high level of PM_2.5_ exposure and low level of PA. The other four groups were group of moderate level of PM_2.5_ exposure and moderate level of PA (Mod 11), group with moderate level of PM_2.5_ exposure and high level of PA (Mod 12), group with low level of PM_2.5_ exposure and moderate level of PA (Mod 21), and group of low level of PM_2.5_ exposure and high level of PA (Mod 22), respectively.

Abbreviations: HR, hazard ratio; CI, confidence interval; PA, physical activity; PM_2.5_, particulate matter with aerodynamic diameter < 2.5 µm.

Table S4. Combined effects of long-term PM_2.5_ exposure levels and physical activity (PA) levels on COPD incidence on multiplicative scales

| **PA level** * | **PM_2.5_ level** * | | | ***P* for interaction** |
| --- | --- | --- | --- | --- |
|  | **High** | **Moderate** | **Low** |  |
| **Self-reported PA level †** |  |  |  | 0.805 |
| Low | 1.000 |  |  |  |
| Moderate |  | 0.926 (0.795, 1.080) | 0.988 (0.844, 1.157) |  |
| High |  | 0.962 (0.824, 1.123) | 0.958 (0.816, 1.124) |  |
| **Self-reported PA level † ‡** |  |  |  | 0.998 |
| Low | 1.000 |  |  |  |
| Moderate |  | 0.993 (0.827, 1.193) | 1.012 (0.837, 1.223) |  |
| High |  | 1.011 (0.841, 1.216) | 0.999 (0.825, 1.210) |  |
| **Objectively-measured PA level †** |  |  |  | 0.918 |
| Low | 1.000 |  |  |  |
| Moderate |  | 0.961 (0.688, 1.343) | 1.054 (0.749, 1.484) |  |
| High |  | 0.866 (0.592, 1.266) | 0.898 (0.611, 1.320) |  |

**†**: Cox proportional hazard model adjusted by age at enrolment, sex, ethnicity, household income, employment status, education, Townsend deprivation index, smoking status, alcohol intake frequency, fruit and vegetable intake, body mass index (BMI) and secondhand smoke exposure.

**‡**: Cox proportional hazard model was further adjusted by baseline lung function and inverse distance to main road.

*: PM_2.5_ exposure levels (low, moderate, and high) were defined by PM_2.5_ tertiles (< 9.48 µg/m^3^, 9.48 to 10.27 µg/m^3^, and ≥ 10.27 µg/m^3^), self-reported PA levels were defined according to a standard scoring criteria of International Physical Activity Questionnaire (IPAQ): low (< 600 MET-min/week), moderate (600 to 3000 MET-min/week), and high (≥ 3000 MET-min/week), and objectively-measured PA levels were defined by objectively-measured PA tertiles [< 24.32 milli-gravity (mg), 24.32 to 30.70 mg, and ≥ 30.70 mg].

Abbreviations: HR, hazard ratio; CI, confidence interval; PA, physical activity; PM_2.5_, particulate matter with aerodynamic diameter < 2.5 µm; COPD, chronic obstructive pulmonary diseases.

Table S5. Associations of PM_2.5_ exposure, self-reported PA levels and COPD incidence further adjusted by baseline lung function, inverse distance to major road, and both

|  | **Original †** | | **Adjusted by Baseline lung function †** | | **Adjusted by Inverse distance to major road †** | | **Adjusted by Both †** **‡** | |
| --- | --- | --- | --- | --- | --- | --- | --- | --- |
|  | **HR (95% CI)** | ***P*** | **HR (95% CI)** | ***P*** | **HR (95% CI)** | ***P*** | **HR (95% CI)** | ***P*** |
| **PA level** * |  |  |  |  |  |  |  |  |
| Low | 1.000 | - | 1.000 | - | 1.000 | - | 1.000 | - |
| Moderate | 0.769 (0.720, 0.820) | <0.0001 | 0.833 (0.770, 0.900) | <0.0001 | 0.782 (0.723, 0.845) | <0.0001 | 0.832 (0.770, 0.899) | <0.0001 |
| High | 0.726 (0.679, 0.776) | <0.0001 | 0.807 (0.745, 0.874) | <0.0001 | 0.737 (0.680, 0.798) | <0.0001 | 0.807 (0.745, 0.874) | <0.0001 |
| Trend test |  | <0.0001 |  | <0.0001 |  | <0.0001 |  | <0.0001 |
| Per 600 MET-min/week increment | 0.987 (0.982, 0.993) | <0.0001 | 0.987 (0.982, 0.993) | <0.0001 | 0.989 (0.982, 0.996) | 0.001 | 0.989 (0.982, 0.996) | 0.001 |
| **PM_2.5_ level** * |  |  |  |  |  |  |  |  |
| Low | 1.000 | - | 1.000 | - | 1.000 | - | 1.000 | - |
| Moderate | 1.050 (0.987, 1.117) | 0.125 | 1.101 (1.023, 1.184) | 0.010 | 1.098 (1.021, 1.182) | 0.012 | 1.102 (1.024, 1.185) | 0.010 |
| High | 1.068 (1.001, 1.140) | 0.045 | 1.091 (1.010, 1.179) | 0.027 | 1.104 (1.021, 1.194) | 0.013 | 1.097 (1.014, 1.186) | 0.021 |
| Trend test |  | 0.046 |  | 0.028 |  | 0.013 |  | 0.021 |
| Per IQR increment | 1.065 (1.032, 1.099) | <0.0001 | 1.058 (1.019, 1.098) | 0.003 | 1.076 (1.034, 1.119) | <0.0001 | 1.068 (1.026, 1.111) | 0.001 |

**†**: The results were fully adjusted by covariates in Model 4: age at enrolment, sex, ethnicity, household income, employment status, education, Townsend deprivation index, smoking status, alcohol intake frequency, fruit and vegetable intake, body mass index (BMI), secondhand smoke exposure, and PA (effect estimation of PM_2.5_) or PM_2.5_ (effect estimation of PA).

**‡**: Cox proportional hazard model was further adjusted by baseline lung function and inverse distance to main road.

*: Self-reported PA levels were defined according to a standard scoring criteria of International Physical Activity Questionnaire (IPAQ): low (< 600 MET-min/week), moderate (600 to 3000 MET-min/week), and high (≥ 3000 MET-min/week).

Abbreviations: PM_2.5_, particulate matter with aerodynamic diameter < 2.5 µm; PA, physical activity; HR, hazard ratio; CI, confidence interval; COPD, chronic obstructive pulmonary diseases.

The bold type represents the statistically significant differences (*p* < 0.05).

Table S6. Combined effects of long-term PM_2.5_ exposure and self-reported physical activity (PA) on COPD incidence further adjusted by baseline lung function, inverse distance to major road, and both

| **PA levels** * | **PM_2.5_ levels (HR, 95% CI)** * **†** | | |
| --- | --- | --- | --- |
|  | **High** | **Moderate** | **Low** |
| **Original** |  |  |  |
| Low | 1.000 | 1.026 (0.909, 1.159) | 0.955 (0.840, 1.085) |
| Moderate | 0.792 (0.711, 0.881) | 0.753 (0.674, 0.840) | 0.747 (0.667, 0.836) |
| High | 0.745 (0.668, 0.831) | 0.736 (0.658, 0.822) | 0.681 (0.607, 0.764) |
| **Adjusted by Both ‡** |  |  |  |
| Low | 1.000 | 1.002 (0.865, 1.162) | 0.906 (0.776, 1.058) |
| Moderate | 0.831 (0.731, 0.945) | 0.827 (0.725, 0.943) | 0.762 (0.665, 0.873) |
| High | 0.804 (0.705, 0.916) | 0.814 (0.713, 0.930) | 0.728 (0.634, 0.835) |

**†**: Cox proportional hazard model adjusted by age at enrolment, sex, ethnicity, household income, employment status, education, Townsend deprivation index, smoking status, alcohol intake frequency, fruit and vegetable intake, body mass index (BMI) and secondhand smoke exposure.

**‡**: Cox proportional hazard model was further adjusted by baseline lung function and inverse distance to main road.

*: Self-reported PA levels were defined according to a standard scoring criteria of International Physical Activity Questionnaire (IPAQ): low (< 600 MET-min/week), moderate (600 to 3000 MET-min/week), and high (≥ 3000 MET-min/week).

Abbreviations: HR, hazard ratio; CI, confidence interval; PM_2.5_, particulate matter with aerodynamic diameter < 2.5 µm; PA, physical activity; COPD, chronic obstructive pulmonary diseases.

The bold type represents the statistically significant differences (*p* < 0.05).

**Table S7. Associations between self-reported PA, long-term PM_2.5_ exposure and COPD incidence by treating all-cause death as a competing risk**

| Levels * | Competing risk analysis **†** (N=266,280) | |
| --- | --- | --- |
|  | HR (95% CI) | *P* |
| Self-reported PA levels |  |  |
| Low | 1.000 | - |
| Moderate | 0773 (0.723, 0.826) | <0.0001 |
| High | 0.714 (0.651, 0.782) | <0.0001 |
| Trend test |  | <0.0001 |
| Per 600 MET-min/week increment | 0.987 (0.981, 0993) | <0.0001 |
| PM_2.5_ levels |  |  |
| Low | 1.000 | - |
| Moderate | 1.052 (0.989, 1.120) | 0.106 |
| High | 1.068 (1.000, 1.140) | 0.049 |
| Trend test |  | 0.050 |
| Per IQR increment (1.27 µg/m^3^) | 1.064 (1.031, 1.098) | 0.0001 |

**†**: Fine-Gray subdistribution hazards were calculated incorporating all-cause death as a competing risk by adjusted covariates in Model 4.

*: PM_2.5_ exposure levels (low, moderate, and high) were defined by PM_2.5_ tertiles (< 9.48 µg/m^3^, 9.48 to 10.27 µg/m^3^, and ≥ 10.27 µg/m^3^), self-reported PA levels were defined according to a standard scoring criteria of International Physical Activity Questionnaire (IPAQ): low (< 600 MET-min/week), moderate (600 to 3000 MET-min/week), and high (≥ 3000 MET-min/week).

Abbreviations: HR, hazard ratio; CI, confidence interval; PA, physical activity; PM_2.5_, particulate matter with aerodynamic diameter < 2.5 µm; COPD, chronic obstructive pulmonary disease.

**Table S8. Associations between self-reported PA, long-term PM_2.5_ exposure and COPD incidence** **after conducting multiple imputations for missing covariates**

| Levels * | Multiple imputation **†**  (N=357,603) | |
| --- | --- | --- |
|  | HR (95% CI) | *P* |
| Self-reported PA levels |  |  |
| Low | 1.000 | - |
| Moderate | 0.788 (0.752, 0.825) | <0.0001 |
| High | 0.765 (0.730, 0.802) | <0.0001 |
| Trend test |  | <0.0001 |
| Per 600 MET-min/week increment | 0.991 (0.987, 0.995) | <0.0001 |
| PM_2.5_ levels |  |  |
| Low | 1.000 | - |
| Moderate | 1.079 (1.031, 1.130) | 0.0011 |
| High | 1.061 (1.012, 1.113) | 0.0138 |
| Trend test |  | 0.021 |
| Per IQR increment (1.27 µg/m^3^) | 1.039 (1.016, 1.062) | <0.0001 |

**†**: Missing values of the baseline covariates were imputed with multivariate imputation via chained equation (MICE). All results were calculated adjusted by covariates in Model 4.

*: PM_2.5_ exposure levels (low, moderate, and high) were defined by PM_2.5_ tertiles (< 9.48 µg/m^3^, 9.48 to 10.27 µg/m^3^, and ≥ 10.27 µg/m^3^), self-reported PA levels were defined according to a standard scoring criteria of International Physical Activity Questionnaire (IPAQ): low (< 600 MET-min/week), moderate (600 to 3000 MET-min/week), and high (≥ 3000 MET-min/week).

Abbreviations: HR, hazard ratio; CI, confidence interval; PA, physical activity; PM_2.5_, particulate matter with aerodynamic diameter < 2.5 µm; COPD, chronic obstructive pulmonary diseases.

**Table S9.** **Interaction between PA and long-term PM_2.5_ exposure on both additive and multiplicative scales after conducting multiple imputations for missing covariates (N=357,603)**

|  | PM_2.5_ level * **†** | | | RERI **‡** **†** | | *P* for interaction § |
| --- | --- | --- | --- | --- | --- | --- |
|  | High | Moderate | Low | Moderate PM_2.5_ level | High PM_2.5_ level |  |
| PA level * |  |  |  |  |  | 0.842 |
| Low | 1.000 | 1.013 (0.932, 1.101) | 0.920 (0.840, 1.008) |  |  |  |
| Moderate | **0.784 (0.730, 0.843)** | **0.783 (0.726, 0.845)** | **0.748 (0.690, 0.811)** | -0.014 (-0.113, 0.084) | 0.016 (-0.082, 0.113) |  |
| High | **0.754 (0.700, 0.811)** | **0.783 (0.725, 0.845)** | **0.712 (0.660, 0.772)** | 0.044 (-0.055, 0.143) | 0.038 (-0.060, 0.137) |  |

**†**: Missing values of the baseline covariates were imputed with multivariate imputation via chained equation (MICE). All results were calculated adjusted by covariates in Model 4. The results were presented by hazard ratio and corresponding 95% confidence intervals (HR, 95% CI).

**‡**: The estimates of RERI were calculated based on the reference group with high level of PM_2.5_ exposure and low level of PA.

§: Likelihood tests was applied to test the significance of interaction term by comparing the model with and without the interaction term.

*: PM_2.5_ exposure levels (low, moderate, and high) were defined by PM_2.5_ tertiles (< 9.48 µg/m^3^, 9.48 to 10.27 µg/m^3^, and ≥ 10.27 µg/m^3^), self-reported PA levels were defined according to a standard scoring criteria of International Physical Activity Questionnaire (IPAQ): low (< 600 MET-min/week), moderate (600 to 3000 MET-min/week), and high (≥ 3000 MET-min/week).

Abbreviations: HR, hazard ratio; CI, confidence interval; RERI, relative excess risk due to interaction; PA, physical activity; PM_2.5_, particulate matter with aerodynamic diameter < 2.5 µm; COPD, chronic obstructive pulmonary diseases.

The bold type represents the statistically significant differences (*p* < 0.05).

Table S10. Baseline characteristics of the excluded and included participants

| **Level** * | **Overall** | **Excluded participants** | **Included participants** |
| --- | --- | --- | --- |
| **Total number** | 502,490 | 236,210 | 266,280 |
| **Age (year)** | 56.53 (8.10) | 57.20 (8.06) | 55.93 (8.08) |
| **Sex** |  |  |  |
| Female | 273,375 (54.40) | 137,989 (57.92) | 135,386 (51.23) |
| Male | 229,114 (45.60) | 100,232 (42.08) | 128,882 (48.77) |
| **Ethnicity** |  |  |  |
| Nonwhite | 29,810 (5.93) | 17,958 (7.60) | 11,852 (4.45) |
| White | 472,680 (94.07) | 218,252 (92.40) | 254,428 (95.55) |
| **PA level** |  |  |  |
| Low | 76,215 (18.94) | 27,113 (19.92) | 49,102 (18.44) |
| Moderate | 164,017 (40.76) | 54,685 (40.18) | 109,332 (41.06) |
| High | 162,136 (40.30) | 54,290 (39.89) | 107,846 (40.50) |
| **PM_2.5_ (ug/m^3^)** | 9.99 (1.06) | 10.07 (1.07) | 9.94 (1.04) |

*: The statistics are shown as mean [standard deviation (SD)] for continuous variables and number (%) for categorical variables.

Abbreviations: PA, physical activity; PM_2.5_, particulate matter with an aerodynamic diameter < 2.5μm.


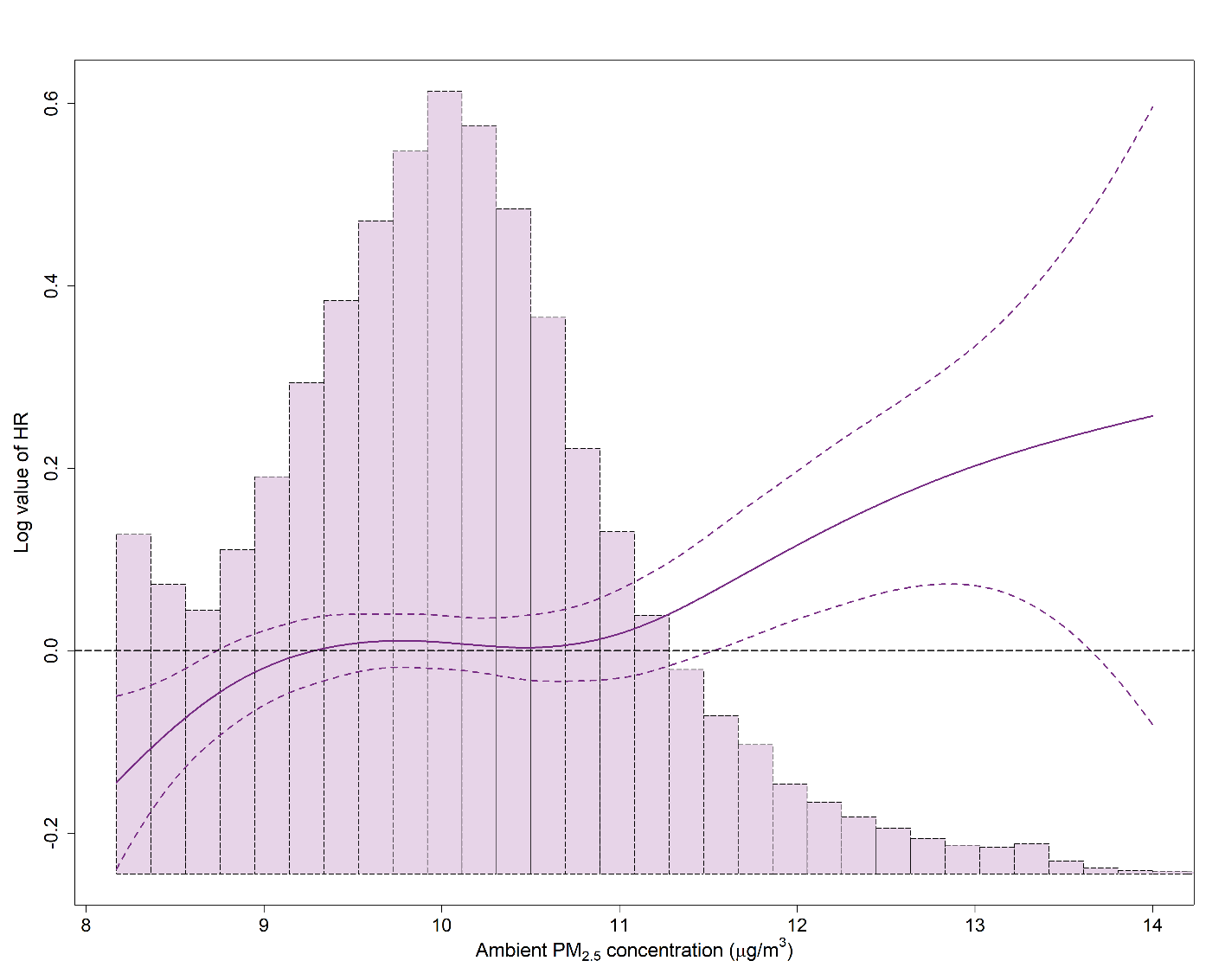


Figure S1. Concentration-response relationship between long-term PM_2.5_ exposure and COPD incidence*

*: Concentration-response relationship between long-term PM_2.5_ exposure [treated as a continuous variable with a degree of freedom (df) of 4] and COPD incidence was examined using a spline term in Cox model, which was adjusted by covariates in Model 4: age at enrolment, sex, and ethnicity, household income, employment status, education, Townsend deprivation index, lifestyle factors (smoking status, alcohol intake frequency, fruit and vegetable intake), body mass index (BMI), secondhand smoke exposure and physical activity level.
